# Length of Acupuncture Training and Structural Plastic Brain Changes in Professional Acupuncturists

**DOI:** 10.1371/journal.pone.0066591

**Published:** 2013-06-19

**Authors:** Minghao Dong, Ling Zhao, Kai Yuan, Fang Zeng, Jinbo Sun, Jixin Liu, Dahua Yu, Karen M. von Deneen, Fanrong Liang, Wei Qin, Jie Tian

**Affiliations:** 1 School of Life Sciences and Technology, Xidian University, Xi’an, Shaanxi, China; 2 The 3rd Teaching Hospital, Chengdu University of Traditional Chinese Medicine, Chengdu, Sichuan, China; 3 Information Processing Laboratory, School of Information Engineering, Inner Mongolia University of Science and Technology, Baotou, Inner Mongolia, China; 4 Institute of Automation, Chinese Academy of Sciences, Beijing, China; Tokai University, Japan

## Abstract

**Background:**

The research on brain plasticity has fascinated researchers for decades. Use/training serves as an instrumental factor to influence brain neuroplasticity. Parallel to acquisition of behavioral expertise, extensive use/training is concomitant with substantial changes of cortical structure. Acupuncturists, serving as a model par excellence to study tactile-motor and emotional regulation plasticity, receive intensive training in national medical schools following standardized training protocol. Moreover, their behavioral expertise is corroborated during long-term clinical practice. Although our previous study reported functional plastic brain changes in the acupuncturists, whether or not structural plastic changes occurred in acupuncturists is yet elusive.

**Methodology/Principal Findings:**

Cohorts of acupuncturists (N = 22) and non-acupuncturists (N = 22) were recruited. Behavioral tests were delivered to assess the acupuncturists’ behavioral expertise. The results confirmed acupuncturists’ tactile-motor skills and emotion regulation proficiency compared to non-acupuncturists. Using the voxel-based morphometry technique, we revealed larger grey matter volumes in acupuncturists in the hand representation of the contralateral primary somatosensory cortex (SI), the right lobule V/VI and the bilateral ventral anterior cingulate cortex/ventral medial prefrontal cortex. Grey matter volumes of the SI and Lobule V/VI positively correlated with the duration of acupuncture practice.

**Conclusions:**

To our best knowledge, this study provides first evidence for the anatomical alterations in acupuncturists, which would possibly be the neural correlates underlying acupuncturists’ exceptional skills. On one hand, we suggest our findings may have ramifications for tactile-motor rehabilitation. On the other hand, our results in emotion regulation domain may serve as a target for our future studies, from which we can understand how modulations of aversive emotions elicited by empathic pain develop in the context of expertise. Future longitudinal study is necessary to establish the presence and direction of a causal link between practice/use and brain anatomy.

## Introduction

“Plasticity is an intrinsic property of the human brain and gives an evolution’s invention to enable the nervous system to escape restrictions of its own genome” [1]. Use is a pivotal factor initiating plastic reorganizations of cortical maps [2]. Indeed, imaging studies have provided compelling evidence that training/enhanced use of a body part or cognitive functions cause structural plastic changes in human central nervous system in the model of dancers [3], sports experts [4] and meditators [5]. These findings consolidated the concept of use-dependent plasticity in humans, according to which changes in cortical maps are dependent on the amount of use that an individual allocates to conform to the requirements of environmental constraints [2].

**Figure 1:**
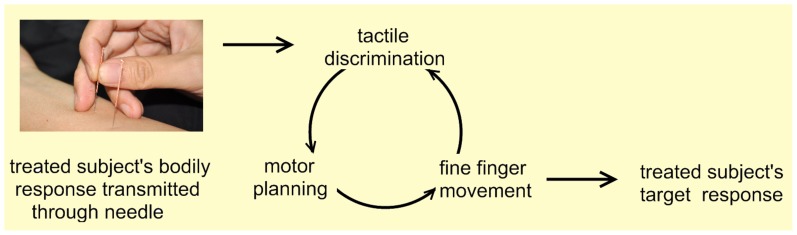
The schematic diagram of tactile-motor procedures during acupuncture manipulation. The schematic diagram for the feedback loop underlying tactile-motor procedures in the process of acupuncture. The patients’ concurrent bodily response to each round of needling manipulation is transmitted to acupuncturist through the fine needle. The acupuncturist distinguishes the subtle difference between the actual tactile sensation and the expected one. The tactile discrimination is followed by motor planning procedure in which the next-step plan of method of needling manipulation, frequency and intensity of rotation is generated. Then, the postural configuration from the motor plan is executed as acupuncturist’s fine and coordinated finger movement over the needles. This feedback loop is repeated until the target response is obtained.

Acupuncturists forms a model par excellence for understanding tactile-motor plasticity, as well as plastic changes in the emotion regulation domain, which was demonstrated in a previous study [6]. Specifically, acupuncture process that relies mainly on the acupuncturists’ tactile feedback to manipulating fingers to generate motor plans, which is to be implemented as fine finger movement (Figure 1). Also, acupuncture genuinely is an invasive procedure inflicting painful treatment to achieve therapeutic effects [7], such as inserting needles into the skull, into the bottom of feet or into the area around canthus, etc. Although this process seems unitary or even automatic for professional acupuncturists, it can be decomposed into several interacting components in sensorimotor and cognitive domains: 1) exceptional tactile discrimination ability which enable the acupuncture practitioners to distinguish subtle dynamic changes of manipulation sensation transmitted through fine needles because a patient’s concurrent physical status and synchronous bodily response to each round of needling manipulation is constantly changing; 2) the ability of making precise adjustment and promptly generating precise motor plans/commands [8]; 3) fine/dexterous motor skills of manipulating fingers; 4) emotion regulation proficiency which is indispensable since acupuncture would induce empathic pain in acupuncturists and massive exposure to such situation would naturally induce negative emotions or personal distress in acupuncturists, which would eventually impair their professionalism [6].

Basically, acupuncturists, a major part of physicians in China, receive intensive training in national medical schools following standardized training protocols [7] and their behavioral expertise are corroborated during long-term clinical practice. It has been shown that, after extensive training/use, behavioral expertise is paralleled by a profound reorganization in brain architecture, characterized by an enlargement of the representations of trained body parts [9]. Our recent study reported functional alterations in the acupuncturist’ brain, however, the question whether or not training effects is parallel by allocations of structural changes in acupuncturists’ brain remains elusive.

In the current study, we firstly verified the behavioral expertise of acupuncturists. Then, we expected to see structural differences between the professional acupuncturists group and the non-acupuncturist group (NA). Accordingly, a voxel-based morphometry (VBM) technique was used to detect the morphological brain changes between a group of professional acupuncturists (N = 22) and NA (N = 22). Further, we would like to see how training duration corresponds to the structural brain changes, therefore, regression analysis between training duration and VBM differences in acupuncturists was conducted.

To our knowledge, this is the first study focusing on the structural plastic changes in professional acupuncturists. Hopefully, our study can provide primary evidence for tactile-motor plasticity as well as the plasticity in the emotion regulation domain.

## Materials and Methods

### 1. Ethics Statement

All research procedures were approved by the West China Hospital Subcommittee on Human Studies and were conducted in accordance with the Declaration of Helsinki. Written informed consent was obtained after the experimental procedures were fully explained.

### 2. Subjects

We investigated 44 healthy and right-handed adult volunteers [10]. The experimental group, consisting of 22 licensed professional acupuncturists (mean age = 28.7±1.4 years, 12 males, education 19.3±1.3 years) was compared with a sample of 22 matched NA (mean age = 28.2±2.3 years, 12 males, education 19.4±1.5 years). All acupuncturists attended national medical schools, receiving 5 years of training with basic acupuncture knowledge, passed the national exam and received a license for acupuncture practice. The mean duration of acupuncture training and practice was 81.0±17.2 months. On the other hand, the control group consisted of working staff in a medical school that also received their degree from the medical school but only did office work and had not worked under any clinical conditions in the past four years. They were included in this study because they had not attended any acupuncture lectures or lectures in related fields, had no experience in acupuncture practice or had no exposure to acupuncture manipulation in any available forms before. We also ensured that no participants possessed proficiency in playing instruments. Subjects reported no past or current neurological, psychiatric, or neuropsychological problems, and did not take drugs or illegal medication prior to or during this study. Written informed consent was obtained after the experimental procedures were fully explained.

### 3. Behavioral Tests

We used three tasks to measure the acupuncturists’ behavioral expertise. Specifically, we examined the subjects’ tactile discrimination ability for the right index finger and thumb, fine motor skill, and emotion regulation ability. In general, for in all three tests, the subjects were seated comfortably in a quiet room with minimal distraction from the surroundings. After the experimental procedures and requirements of the tasks were explained to the subjects by the same experimenter, the subjects were required to repeat the procedures and demands of the tasks to the experimenter to ensure all details were explicitly comprehended. These tests did not begin until the experimenter was sure that all procedures were precisely understood by all of the participants.

#### a) Test on tactile discrimination ability

Tactile spatial acuity is a reliable indicator of somatosensory system function. Previous studies have proved that tactile spatial acuity is highly correlated with subjective tactile perceptual integrity in a broad range of subject populations [11], [12], [13]. A well-established and reproducible measure of tactile spatial acuity is the psychophysical spatial discrimination threshold (SDT) for the grating orientation discrimination task [12], [13], [14]. SDT was assessed using Johnson–Van Boven–Phillips domes (Med-Core, St. Louis) at the fingertip [14].

In the current study, we used the grating orientation discrimination task to evaluate the subjects’ tactile discrimination ability of the right index finger and thumb. The fingers to be tested were immobilized through double-sided tape affixed to the dorsal aspect of the finger and floor of the fixture to prevent exploratory movements. Grating domes were pressed manually onto the palmar surface of the distal phalanx of the thumb and index finger of the right hand using a spring-loaded apparatus with a spring-loaded force of 1.5±0.2 N, which was adapted following the procedure of a former study [15]. Gratings were applied with the ridges oriented either along or across the long axis of the finger and subjects verbally reported the orientation of the grating as ‘along’ or ‘across’. During this experiment, subjects were blindfolded and seated comfortably. The experimenter was visually cued to manually position [15], then released the gratings to the fingertip and maintained static for 1 second as signaled by a computer-driven timing mechanism [16]. The subsequent experimental block on each finger consisted of 40 trials without feedback. Participants received a 15 s break after every 20 trials, and a 1 min break between fingers. Each trial consisted of two sequential stimulus presentations (inter-stimulus interval, 2 s) with gratings of identical groove width but differing 90° in orientation, i.e. either parallel (vertical) to or transverse (horizontal) to the long axis of the finger. The stimulus order was chosen randomly. Subsequently, for each finger of each subject, dome gratings with progressively finer spatial periods were used until performance was at or below threshold levels (75% correct responses) [15], [17], [18].The grating sizes yielded the SDTs for two fingers of each individual.

The SDT was the outcome for this task.

#### b) Test on fine motor skill

An in-house motor task was used in this test to assess the subjects’ fine motor skill. The task was designed according to the core kinetic feature of acupuncture manipulation. In the standardized training protocol and acupuncturists’ clinical practice, the most basic requirement is that one should rotate the needle quickly and rhythmically, so that therapeutic effects can be optimally elicited [7]. In this sense, in our motor task, the subjects were required to rotate the needle as quickly as possible while making maximal effort to keep the rotation angle of each round within the range of 90°-180°in a pre-determined period, i.e. 30 seconds. A round was defined as from the starting position to the end position where the direction of the rotation changed.

We developed an integrated system enabling on-line recording of needle rotation. An acupuncture needle (2.5 inch in length and 0.25 in diameter) was inserted 2 inches deep into pork. Among all available materials, we chose pork because the resistance delivered to the manipulating hand could best replicate that of the human body. A marker was equipped at the end of the needle to mark the rotation angle for data analysis. A high speed camera (24frames/sec) recorded the whole process of needle rotating. An in-house computer program was developed to calculate the number of rotations for each round, eliminating the rounds whose rotation angles fell out of the required range. Only rounds meeting our criteria were counted. The subject was asked to rotate the needle using the tip of the right index finger and thumb. The palm of the right hand had to stay in that position (almost) vertical to the table surface, but not parallel to the table surface. This needling posture is the most common one in a clinical situation and therefore is the basic needling technique for acupuncturists. No right upper limb parts (e.g. palm, elbow or arm) were allowed to touch the table. Before the task, participants were seated before a table where our system was placed. After the participants adjusted themselves to the most comfortable needling position, they started the process. To ensure the reliability of the measurement, each subject was asked to perform the task three times. For each time, the number of rounds meeting the criteria was counted. The three counts were averaged for each subject. The average number of needle rotations was the outcome for this task.

We evaluated the test-retest reliability of our motor test. The intra-class correlation (ICC), a common measure of test-retest reliability, was employed to assess the stability between multiple measures of the same concept [19], [20], [21]. The ICC value is between -1.0 to 1.0. An ICC <0.41, from 0.41–0.59, from 0.6–0.75, or >0.75 indicates a poor, fair, good, or excellent reliability respectively [20]. In our study, ICC >0.5 was used as the threshold to determine whether the test is reliable or not according to the previous literature [22]. The SPSS 18.0 was used to compute the ICC.

#### c) Test on emotion regulation proficiency

Acupuncturists’ daily clinical practice naturally leads to empathic pain, which would cause a negative emotional response, such as unpleasantness or distress [23]. There must be a certain central mediating mechanism which helps them cope with adverse daily events and prevents negative emotions from impairing their ability to heal or be of assistance [6]. This test aimed to assess the acupuncturists’ emotion regulation proficiency. A unpleasantness rating task used in a previous study was employed [6].

One week before the task, participants filled out a series of self-report dispositional measures, including the situational pain questionnaire (SPQ) that assessed sensitivity to pain [24], the Emotional Contagion Scale(ECS) that measured the susceptibility to others’ emotions [25], and the interpersonal reactivity index (IRI) [26]. During the task, all subjects were shown 120 visual stimuli (120 jpeg files). These stimuli consisted of pictures of different body parts of both painful and neutral situations [6]. Pictures were scenarios encountered in daily clinical practice. In half of the stimuli, the body parts were touched by a Q-tip (non-painful situations), and in the other half they were pricked by an acupuncture needle (painful situations). All body parts were chosen to be appropriate acupuncture sites with the assistance of an acupuncture physician with over ten years of clinical experience. The visual stimuli were delivered using the computer program E-prime 2.0 (Psychology Software Tools, Inc.). The sequence of images was randomized. Each image was displayed for 4 seconds, and rating for unpleasantness lasted 2 seconds. Participants were asked to focus on the images shown on the screen and began to score only after the cue for scoring appeared. The screen for scoring read ‘*How intense is the unpleasantness felt by you now?*’. Participants responded on a 10-point Likert scale ranging from 1 to 10, where 10 referred to the extreme unpleasantness, and 1 referred to no effect.

The ratings for unpleasantness were averaged across neutral and negative stimuli respectively for each subject, rather than variations in body parts that were stimulated. The ratings for both negative and neutral stimuli were the outcomes for this task, since they reliably reflected the subjects’ emotion regulation ability [27].

### 4. MRI Data Acquisition

Imaging data was performed on a 3T Siemens scanner (Allegra; Siemens Medical System) at the Huaxi MR Research Center, West China Hospital of Sichuan University, Chengdu, China. A standard birdcage head coil was used, along with restraining foam pads to minimize head motion and to diminish scanner noise. The axial 3D T1-weighted images were obtained using an MPRAGE sequence with the following parameters: TR = 1900 ms; TE = 2.26 ms; flip angle = 90°; in-plane matrix resolution = 256×256; slices = 176; field of view = 256×256 mm^2^; voxel size = 1×1×1 mm^3^.

### 5. Voxel-Based Morphometry

Before analysis, the structural images were examined by a professional radiologist to exclude the possibility of clinically silent lesions for all of the participants. Structural data was analyzed with FSL-VBM, a voxel-based morphometry style analysis [28], [29] carried out using FSL4.1 [30] (www.fmrib.ox.ac.uk/fsl/). First, all T1 images were brain-extracted using the brain extracting tool (BET) [31]. Next, tissue-type segmentation was carried out using FMRIB’s automated segmentation tool (FAST) V4.1 [32]. The resulting grey-matter partial volume images were then aligned to MNI152 standard space using the affine registration tool FMRIB’s Linear Image Registration Tool (FLIRT) [33], [34], followed by nonlinear registration using FMRIB’s Nonlinear Image Registration Tool (FNIRT) [35], which uses a b-spline representation of the registration warp field [36]. The resulting images were averaged to create a study-specific template, to which the native grey matter (GM) images were then non-linearly re-registered. The registered partial volume images were then modulated (to correct for local expansion or contraction) by dividing by the Jacobian of the warp field.

The modulated segmented images were then smoothed with an isotropic Gaussian kernel with a sigma of 3 mm (analogous to a 7 mm FWHM). Analysis of covariance (ANCOVA) was employed with age, gender, education status effects and total intracranial volume as covariates. Finally, voxel-wise GLM was applied using permutation-based non-parametric testing with 5000 random permutations, correcting for multiple comparisons across space. Results were considered significant at p<0.01, corrected for multiple comparisons.

To better identify the anatomical region of the cerebellum, cerebellar cluster localization was determined using the probabilistic atlas [37], [38] of the cerebellum with MRIcroN (http://www.cabiatl.com/mricro/mricron/). For display purposes, statistical maps regarding the cerebral regions were superposed on the ‘ch2bet’ template in MRIcro (http://www.cabiatl.com/mricro/mricro/) and the cerebellar regions were overlaid on a spatially unbiased atlas template of the cerebellum [39] using MRIcroN, following the current consensus in nomenclature [40], [41].

### 6. Regression Analysis

For the regions where acupuncturists showed significantly different gray matter volumes over the controls, the gray matter volumes of these areas were extracted, averaged and regressed against the duration of acupuncture practice. Additionally, to detect the correspondence between central representations and behavioral expertise, the gray matter volumes of these areas were extracted, averaged and regressed against the measurement of behavioral expertise. Results were considered significant at *p<0.05* (multiple correction using Bonferroni test).

## Results

### 1. Results of Behavioral Tests

#### a) Results of tactile discrimination ability test

As shown in Table 1, the two sample *t*-test revealed that the acupuncturists had a significantly lower SDT than that of the control group for both fingers, indicating better spatial acuity (*two sample t-test, p<0.05 for the index finger and the thumb*). The results verified the acupuncturists’ tactile discrimination proficiency.

**Table 1:** Measure of fine motor skill for fingers and spatial discrimination threshold in two groups.

	Experts(n = 22)		NA(n = 22)		two sample t (Experts vs. NA)	
Task	Mean	SD	Mean	SD	*p* value	*t* value
NoR*	90.1	6.9	46.1	7.5	2.73E-23	20.2501
SDT*(Indext)	0.93	0.23	1.11	0.18	0.0065	−2.8627
SDT*(Thumb)	1.16	0.18	1.33	0.12	6.60E-04	−3.6795

NoR: number of rotations; SDT: spatial discrimination threshold; SD: standard deviation;

* **denotes the item that shows significant difference between the two groups (** ***p<0.05*** **).**

#### b) Results of fine motor skill test

The intra-class correlation (ICC) coefficient was used to evaluate the test-retest reliability of our motor test. The ICC was 0.95, better than the 0.5 threshold [22]. This indicates that our test is reliable and provides stable measurement.

Then we evaluated whether acupuncturists outperformer the NA in this test. The results displayed that the acupuncturists achieved 90.1±6.9 rounds of rotations as compared to 46.1±7.5 of non-acupuncturists (*two sample t-test, p<0.05*), as shown in Table 1. The results verified that the acupuncturists had better fine motor skills compared to NA.

#### c) Results of the emotion regulation proficiency test

The analyses of the dispositional measures revealed no differences between the two groups in terms of ECS, SPQ and each sub-domain of IRI scores using a *two sample t-test* (*see* *Table 2* *for detailed p and t values*). The detailed information is summarized in Table 2. Results of the two sample *t*-test showed that unpleasantness ratings for neutral stimuli did not differ between the two groups (*two sample t-test, p = 0.39, t = −0.87*), which was consistent with previous conclusions [6], whereas for negative stimuli, unpleasantness ratings were significantly lower in the acupuncturists group (Table 2). The results demonstrated that the acupuncturists had better emotion regulation ability than that of the controls.

**Table 2:** Dispositional measurement of empathy and ratings of unpleasantness in two groups.

	Experts(n = 22)		NA(n = 22)		two sample t (Experts vs. NA)	
Task	Mean	SD	Mean	SD	p value	t value
**ECS**	26.5	3.8	26.6	4.3	0.94	−0.07
**SPQ**	5.8	0.5	5.6	0.8	0.38	0.89
**IRI(PT)**	18.4	3.7	18.2	3.1	0.86	0.17
**IRI(EC)**	21.1	3.2	20.4	3.6	0.45	0.76
**IRI(PD)**	11.8	3.5	12.5	4.1	0.76	−0.31
**IRI(FS)**	17.5	4.1	16.9	3.7	0.65	0.46
**Unpleasantness** *	1.6	0.7	6.1	0.9	1.06E-21	−18.38

ECS: emotional contagion scale; SPQ: situational pain questionnaire; IRI: interpersonal reaction index; PT: perspective taking; EC: empathic concern; PD: personal distress; FS: fantasy; SD: standard deviation;

* **denotes the item that shows significant difference between the acupuncturists and NA (** ***p<0.05*** **).**

### 2. VBM Results

**Figure 2:**
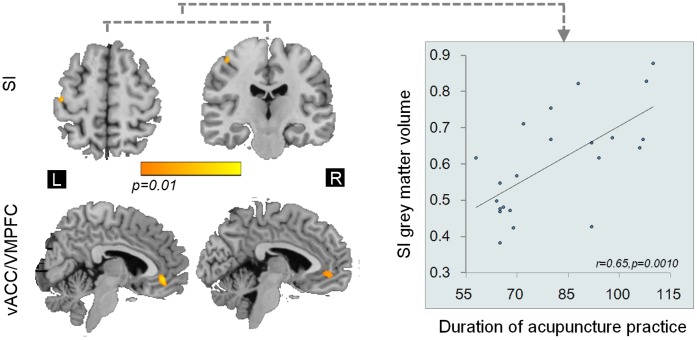
Cerebral VBM differences between groups (*p<0.01, corrected*) and regression analysi*s (p<0.05, Bonferroni corrected)*. The GMV differences in cerebral regions between groups. Positive linear correlations were found between GMV in the left SI and the duration of acupuncture practice. All images are shown as (1-p) corrected *p*-value images at the threshold of *p*<0.01, corrected. The corresponding *t* values are provided. The vACC/VMPFC was displayed in the sagittal view, and SI in the axial view (on the left side) and the coronal view (on the right side) using MRIcro. vACC/VMPFC: ventral anterior cingulate cortex/ventral medial prefrontal cortex; primary somatosensory cortex: SI.

**Figure 3:**
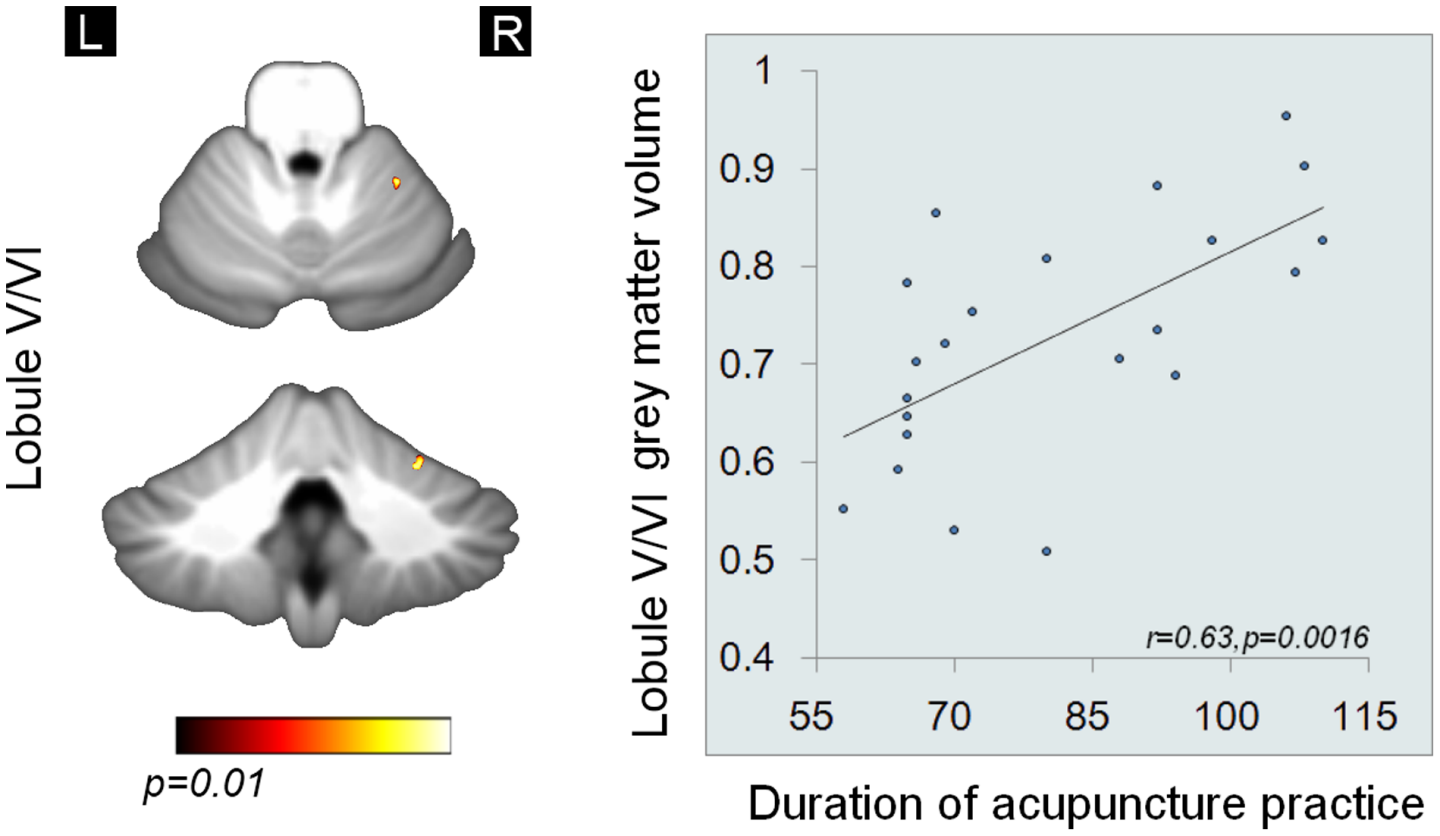
Cerebellar VBM differences between groups (*p<0.01, corrected*) and regression analysis (*p<0.05, Bonferroni corrected)*. The GMV differences in cerebellar regions between groups. Positive linear correlations were found between GMV in the right lobule V/VI and the duration of acupuncture practice. All images are shown as (1-p) corrected *p*-value images at the threshold of *p*<0.01, corrected. The corresponding t values are provided. The Lobule V/VI was displayed in the axial view (the upper figure) and the coronal view (the lower figure) using MRIcroN.

Regional grey matter volume changes were assessed using VBM. Significantly larger grey matter volumes (*p*<0.01) were found in the acupuncturists as compared to NA in a subset of cerebral and cerebellar regions, i.e. the left primary somatosensory cortex (SI), the right lobule V/VI and the bilateral ventral anterior cingulate cortex/the vetral medial prefrontal cortex (vACC/VMPFC) after controlling for potential confounding variables including age, gender effects, level of education and total intracranial volume (shown in Figure 2, Figure 3 and Table 3). Significant grey matter volume (GMV) differences were found exclusively in these regions.

**Table 3:** Significant grey matter volume differences in the acupuncturists group (*p<0.01, corrected*).

	Hemisphere	MNI Coordinates (cluster maxima)			*Peak t-stat*
		x	y	z	
vACC/VMPFC	L	−1	39	−7	6.42
	R	3	47	−7	6.37
SI	L	−44	−20	59	6.53
Lobule V/VI	R	26	−47	−24	6.60

vACC: ventral anterior cingulate cortex; VMPFC: ventral medial prefrontal cortex; SI: primary somatosensory cortex; L, left; R, right.

### 3. Results of Regression Analysis

GMV of the left SI and the right V/VI positively correlated with the duration of acupuncture practice (*p<0.05, Bonferroni corrected*), after regressing out potential confounding variables of age, gender and level of education in our analysis (Figure 2 and Figure 3). While the correlations/anti-correlations between GMV of left SI, the right V/VI and the bilateral vACC/VMPFC and measurement of behavioral expertise were not found. Specifically, we have done 3×5 = 15 corrections for regression analysis (3 ROIs vs. duration of acupuncture practice, outcomes for tactile ability test, i.e. SDT for the two fingers, outcomes for motor task and unpleasantness ratings).

## Discussion

In the current study, we initiated to investigate the structural brain differences between acupuncturists and NA. Firstly, our behavioral analysis verified the behavioral expertise of acupuncturists in tactile-motor and emotion regulation domains (Table 1 and Table 2). The VBM results showed that the acupuncturist group, as compared to NA, had larger GMV in the left SI, secondary motor areas, i.e. the right V/VI, and the bilateral vACC/VMPFC. Our findings confirmed the notions that any manipulation which produces an enduring change in behavior leaves an anatomic footprint on the brain [42]. We also revealed the positive correlation between the GMV of the left SI (Figure 2) and cerebellar V/VI and the duration of acupuncture practice (Figure 3). We suggest that our study may also provide preliminary evidence for neuroanatomical substrates subserving professional acupuncturists’ extraordinary skills.

Our study demonstrated larger GMV in the cortical representation of hand in the left SI cortex. This may be linked to acupuncturists’ exceptional tactile discrimination ability (Table 1). Indeed, tactile discrimination ability is especially crucial for acupuncturists. Acupuncture aims to elicit unique bodily responses in patients, which is characterized as signified but extremely subtle tightness around the needle and the tactile information is used by acupuncturists to characterize optimal therapeutic effects [7]. However, each round of needle manipulation induces a distinctive bodily response in patients because the patients’ concurrent mental state and physical responses to needling are dynamic. In this sense, it is necessary for acupuncturists to decide whether the expected sensation is achieved by carefully discriminating the tactile stimulus delivered to the manipulating digits through the fine needle. The region reported here is known to play an important role in perception of touch [43]. A study applying repetitive transcranial magnetic stimulation over the cortical hand-finger representation of the primary somatosensory cortex revealed a close link between the cortical enlargement and improvement in tactile perception function of the fingers [44], whereas declined tactile perception function was concomitant with shrunken primary somatosensory cortical maps [2]. On one hand, the tactile function is enhanced on the basis of usage [45] and use is a major factor driving plasticity of cortical maps [2]. On the other hand, previous reports further suggested that enrichment in the afferent sensory input could induce cortical morphometry changes in the adult human brain [46]. In our case, the acupuncturists’ daily practice exclusively depends on this sensory modality and the function is extensively used [7], which in return also permits enhanced sensory stimulation in this modality. Therefore, we suggest that larger contralateral representations in the primary somatosensory cortex of the right hand may support the acupuncturists’ extraordinary ability in perceiving subtle tactile information, eventually facilitating their tactile discrimination ability.

We reported larger GMV in the right lobule V/VI in acupuncturists (Figure 3 and Table 3), which may be related to acupuncturists’ extraordinary motor skills for fingers. The acupuncturists’ fine motor skill for fingers is important, since it ultimately determined whether or not optimal clinical outcomes could be achieved. Our behavioral test verified that acupuncture had exceptional fine motor skills (Table 1). The structural changes in this region fits particularly well with the well-established cerebellar involvement in unimanual finger movement control [47], [48]. Specifically, cerebellum has been considered a region largely involved in motor learning [49]. Previous studies using subjects with unimanual finger motor proficiency implicated that skill-relevant functional/structural brain mapping changes occurred in the ipslateral lobule V/VI [50], [51]. Therefore, we suggested that the larger GMV of the right lobule V/VI was likely to support the acupuncturists’ fine motor skill, in parallel, the structural changes may also serve to facilitate performance retention [1].

The regression analysis demonstrated positive correlation between the GMV of the SI vs. duration of acupuncture practice and the GMV of the right lobule V/VI vs. duration of practice. This is consistent with former observations that long-lasting and exceptional usage of the fingers results in the development of outstanding sensorimotor skills and results in expansions of the cortical finger representations and GMV changes of sensorimotor domain might be developed as a function of time [2]. These also imply that the duration of training was potentially an important variable determining the amount of cortical changes.

Acupuncture procedure substantially involves inflicting painful treatment process to patients to achieve therapeutic effect. Acupuncturists have to be massively exposed themselves to such situations each day during their clinical practice. This painful procedure should naturally induced intensive and prominent aversive empathic responses [6], which would probably lead to anxiety and personal distress [23]. Such response would bias their professional help. But, it seems acupuncturists do not suffer from this, as evidenced significantly lower unpleasantness ratings (Table 2). These differences are not likely to be attributed to depositional or emotion contagion because these personality traits did not differ in the two groups (Table 2). It was also unlikely that this difference was due to attention demands because both groups performed similarly on the continuous performance task. Therefore, we proposed there should be a certain emotion regulation mechanism facilitating acupuncturist. Accordingly, we found a larger GMV in the bilateral vACC/VMPFC in the acupuncturist group in the current study. A number of functional, behavioral, and lesion studies have provided evidence that the region is involved in modulation or inhibition of the emotional response [52], [53], [54]. Specifically, this region is in association with suppressing or reappraising negative emotional stimuli and with suppressing the influence of negative emotional stimuli on subsequent behavior [55]. Also, a recent report stated that increased GMV in this region was associated with enhanced emotion regulation control performance after long-term meditation training [5]. In contrast, pathological data demonstrated the dysfunction in emotion regulation was largely associated with decreased GMV in this region [56], [57]. Taken together, we suggest that larger GMV in the vACC/VMPFC is highly likely to be associated with the acupuncturists’ emotion regulation expertise. But, the specific nature of these underlying correlates (e.g., possibly enhanced neuropil, neuronal size, number, and density, size, and/or a particular wiring pattern of neuronal connections in meditators) remains to be established in future studies.

Moreover, no correlation/anti-correlation was found between the GMV of vACC/VMPFC and the duration of acupuncture practice in acupuncturists. Given that emotion regulation was a higher-order cognitive function, we suggested that it was probable that structural underpinnings of this function do not necessarily develop over the same time course with low order sensorimotor functions [58], [59].

### Conclusions

The present findings demonstrate that long-term acupuncture training occurring in adulthood is associated to structural modifications not randomly distributed, but likely to be related to the specific features of acquired skills. In conclusion, acupuncturists represent a useful group to study human brain structural plasticity in tactile-motor and emotion regulation dimensions. On one hand, we suggest our findings may have ramifications for tactile-motor rehabilitation. On the other hand, our results in emotion regulation domain may serve as a target for our future studies, from which we can understand how modulations of aversive emotions elicited by empathic pain develop in the context of expertise. To a greater extent, we hope that this study may help to understand the behavioral-brain inter-connection, which in return may facilitate occupational skill acquisition [60] and will guide to develop new naturalistic training strategies during adulthood in the future [61].

We understand that a major concern in neuroplasticity research is how developmental origin, training and genetic factor interact with each other, ultimately influencing neuroplasticity. Therefore, we should take cautions when interpreting the current findings, since we cannot exclusively account the differences found in this study to the experience/training alone. To this end, longitudinal experimental design is appropriate to definitively link the structural changes and the factor of use/training. But, our currently available data is not feasible for this purpose. Nevertheless, we are optimistic to suggest the potency of taking our results as the basis for further studies. During the following two years, a series of experiments using longitudinal design, with larger sample size, even with subjects from the same family, are to be carried out, to make stronger inferences about the impact of training *per se*. We do hope to answer this question in the long run.
